# Examining barriers to antiretroviral therapy initiation in infants living with HIV in sub‐Saharan Africa despite the availability of point‐of‐care diagnostic testing: a narrative systematic review

**DOI:** 10.1002/jia2.26284

**Published:** 2024-07-05

**Authors:** Chikondi Isabel Joana Chapuma, Doreen Sakala, Maggie Nyirenda Nyang'wa, Mina C. Hosseinipour, Nyanyiwe Mbeye, Mitch Matoga, Moses Kelly Kumwenda, Annastarsia Chikweza, Alinane Linda Nyondo‐Mipando, Victor Mwapasa

**Affiliations:** ^1^ Malawi Liverpool Research Programme Blantyre Malawi; ^2^ Kamuzu University of Health Sciences Blantyre Malawi; ^3^ University College London London UK; ^4^ University of North Carolina Project Lilongwe Malawi

**Keywords:** HIV, infant, diagnosis, antiretroviral therapy, point‐of‐care, sub‐Saharan Africa

## Abstract

**Introduction:**

Antiretroviral therapy (ART) initiation in infants living with HIV before 12 weeks of age can reduce the risk of mortality by 75%. Point‐of‐care (POC) diagnostic testing is critical for prompt ART initiation; however, despite its availability, rates of ART initiation are still relatively low before 12 weeks of age. This systematic review describes the barriers to ART initiation in infants before 12 weeks of age, despite the availability of POC.

**Methods:**

This systematic review used a narrative synthesis methodology. We searched PubMed and Scopus using search strategies that combined terms of multiple variants of the keywords “early infant initiation on antiretroviral therapy,” “barriers” and “sub‐Saharan Africa” (initial search 18th January 2023; final search 1st August 2023). We included qualitative, observational and mixed methods studies that reported the influences of early infant initiation on ART. We excluded studies that reported influences on other components of the Prevention of Mother to Child Transmission cascade. Using a deductive approach guided by the updated Consolidated Framework of Implementation Research, we developed descriptive codes and themes around barriers to early infant initiation on ART. We then developed recommendations for interventions for the identified barriers using the action, actor, target and time framework from the codes.

**Results:**

Of the 266 abstracts reviewed, 52 full‐text papers were examined, of which 12 papers were included. South Africa had most papers from a single country (*n* = 3) and the most reported study design was retrospective (*n* = 6). Delays in ART initiation beyond 12 weeks in infants 0–12 months were primarily associated with health facility and maternal factors. The most prominent barriers identified were inadequate resources for POC testing (including human resources, laboratory facilities and patient follow‐up). Maternal‐related factors, such as limited male involvement and maternal perceptions of treatment and care, were also influential.

**Discussion:**

We identified structural barriers to ART initiation at the health system, social and cultural levels. Improvements in the timely allocation of resources for POC testing operations, coupled with interventions addressing social and behavioural barriers among both mothers and healthcare providers, hold a promise for enhancing timely ART initiation in infants.

**Conclusions:**

This paper identifies barriers and proposes strategies for timely ART initiation in infants.

## 1 INTRODUCTION

Sub‐Saharan Africa (SSA) harbours the highest burden of HIV and has registered the lowest progress in paediatric antiretroviral therapy (ART) treatment coverage at 74% (<95% global target) [1]. In infants exposed to HIV, interventions before 12 weeks are associated with a reduction in the risk of morbidity and mortality by 75% [2, 3]. Therefore, the World Health Organization (WHO) introduced the Early Infant Diagnosis (EID) programme to accelerate the initiation of antiretrovirals (ART) among infants 0–12 months of age who are either exposed or living with HIV. EID involves early virological testing of infants from 6 weeks of age to enable early identification of infants living with HIV as a first step for initiating them on ART [4].

There are several known barriers and facilitators for ART initiation coverage in infants living with HIV. For example, a long turnaround time (TAT) between testing and receipt of results is associated with low ART coverage, because it results in a substantial loss to follow‐up of infants exposed to HIV [5]. For instance, in Malawi, the loss to follow‐up rate is 10% at 12 weeks of age [6]. Point‐of‐care (POC) testing expedites HIV diagnosis [7, 8, 9]. Early infant diagnosis facilitates early ART initiation [10]. Clinical studies have shown that using GeneXpert DNA‐PCR POC testing may reduce the TAT to 1–10 days [11]. In South Africa, the implementation of POC testing has significantly enhanced the delivery of virological test results, with 80% of HIV‐exposed infants now receiving their results [12]. However, a barrier for POC is the high burden of disease. For example, in South Africa, primary healthcare with high clinic attendance and limited laboratory capacity results in some clients being sent back home to get their results on a later day, risking losing the patients to follow‐up [13]. In Malawi, two WHO pre‐qualified POCs, Alere q and Cepheid‐dried blood spots (DBS) have been piloted [11, 14]. Cepheid DBS was rolled out in most Malawian district hospitals in 2018. Further, to improve the TAT of using a Cepheid‐DBS protocol, a previous study evaluated the feasibility and acceptability of Cepheid‐whole blood (WB) at Mulanje district in Malawi [11]. In this study, they found that using a Cepheid‐WB protocol is a facilitator for ART initiation coverage (caregivers received their virological results in 5.34, IQR 2.45, 10.19 hours) [11]. However, it is still not well understood why despite installing POCs in some sub‐Saharan health facilities, TAT remains long (up to 24 days), hence ART initiation for infants is delayed.

This narrative systematic review applied the updated Consolidated Framework of Implementation Research (CFIR) [12]. The CFIR is a meta‐theoretical framework comprising of 39 constructs across five major domains, namely intervention characteristics, inner setting, outer setting, individuals involved and implementation process [15]. It delineates the behaviours that could potentially serve as barriers to an intervention and provides a framework for devising improvement strategies (e.g. employing the Action, Actor, Context, Target and Time framework). Presently, there is a dearth of insights into the barriers and potential strategies for early infant ART initiation in contexts where POC testing is being implemented, but targets are still unmet. This narrative systematic review aims to describe the barriers to ART initiation in infants living with HIV before 12 weeks of age, despite the availability of POC diagnostic testing.

## 2 METHODS

This narrative systematic review was carried out following the Preferred Reporting Items for Systematic Reviews (registered with Prospero ID, CRD42023312174). The search strategy, quality measures, inclusion and exclusion criteria, quality assessment and strategy for data synthesis were prespecified by HIV specialists, paediatricians, social scientists and epidemiologists. This systematic review employed a thematic synthesis methodology for qualitative systematic reviews. We synthesized findings from qualitative studies, observational studies and mixed methods studies to generate common themes.

### 2.1 Search strategy, inclusion and exclusion criteria

We searched PubMed and Scopus using search strategies that combined terms relating to “early infant initiation on antiretroviral therapy,” “barriers” and “sub‐Saharan Africa” (Table S1) (initial search on the 18th January 2023 and final search on the 1st August 2023). We included qualitative studies, observational studies (cross‐sectional, cohort and longitudinal) and mixed methods studies that reported influences of early infant initiation on ART in the SSA region for triangulation (Table S2). We excluded studies that reported influences on early infant diagnosis, studies not from SSA and all the other components of the Vertical Transmission (VT) cascade (Table S3). Though these components are interconnected parts of the overall care cascade, they are independent. Therefore, the exclusion of EID and the other components of the VT are intentional to focus specifically on early infant initiation. We did not apply a filter for time and language.

### 2.2 Study selection and data extraction

CIJC and DS independently screened abstracts and retrieved eligible articles for full‐text review and extracted insights on barriers to early infant initiation on ART. Key outcomes and main results were summarized in a pre‐designed table. CIJC and DS extracted the data relating to author, country in SSA, year, study design and outcome measures into Microsoft Excel. In the data extraction process, both reviewers (CIJC and DS) independently reviewed all the papers using the Rayyan application. After this initial independent extraction, they convened to discuss their findings. During these discussions, any disagreements in the extracted data were addressed, and efforts were made to reach a consensus. In cases where consensus could not be reached, a third reviewer (MNN) was involved to resolve conflicts.

### 2.3 Insight analysis and behavioural specification

In conducting the insight analysis and behavioural specification mapping exercise, CIJC and DS utilized a systematic and structured approach. This involved several key steps. Firstly, a comprehensive list of barriers was compiled. Then, using a deductive approach guided by the updated CFIR, CIJC and DS developed the descriptive codes and themes of barriers to early infant initiation on ART. Subsequently, a data extraction sheet, incorporating Action‐Actor‐Context‐Target‐Time (AACTT) variables, was exported to NVIVO software (version 12) for organization and analysis. The application of AACTT constructs specifically tailored to barriers in early infant initiation on ART to proposed interventions. Differences in insight extractions were also resolved by MNN. A presentation to the Malawi‐HIV‐Implementation Research Science coalition was made for validation of the insights and proposed interventions.

### 2.4 Quality assessment

The two authors also performed evaluations of the single studies using the Strengthening the Reporting of Observational Studies in Epidemiology (STROBE) Criteria; a checklist for adequate reporting for observational studies. The quality of qualitative studies was performed using the Critical Appraisal Skills Programme Qualitative Research Checklist (CASP).

## 3 RESULTS

**Figure 1:**
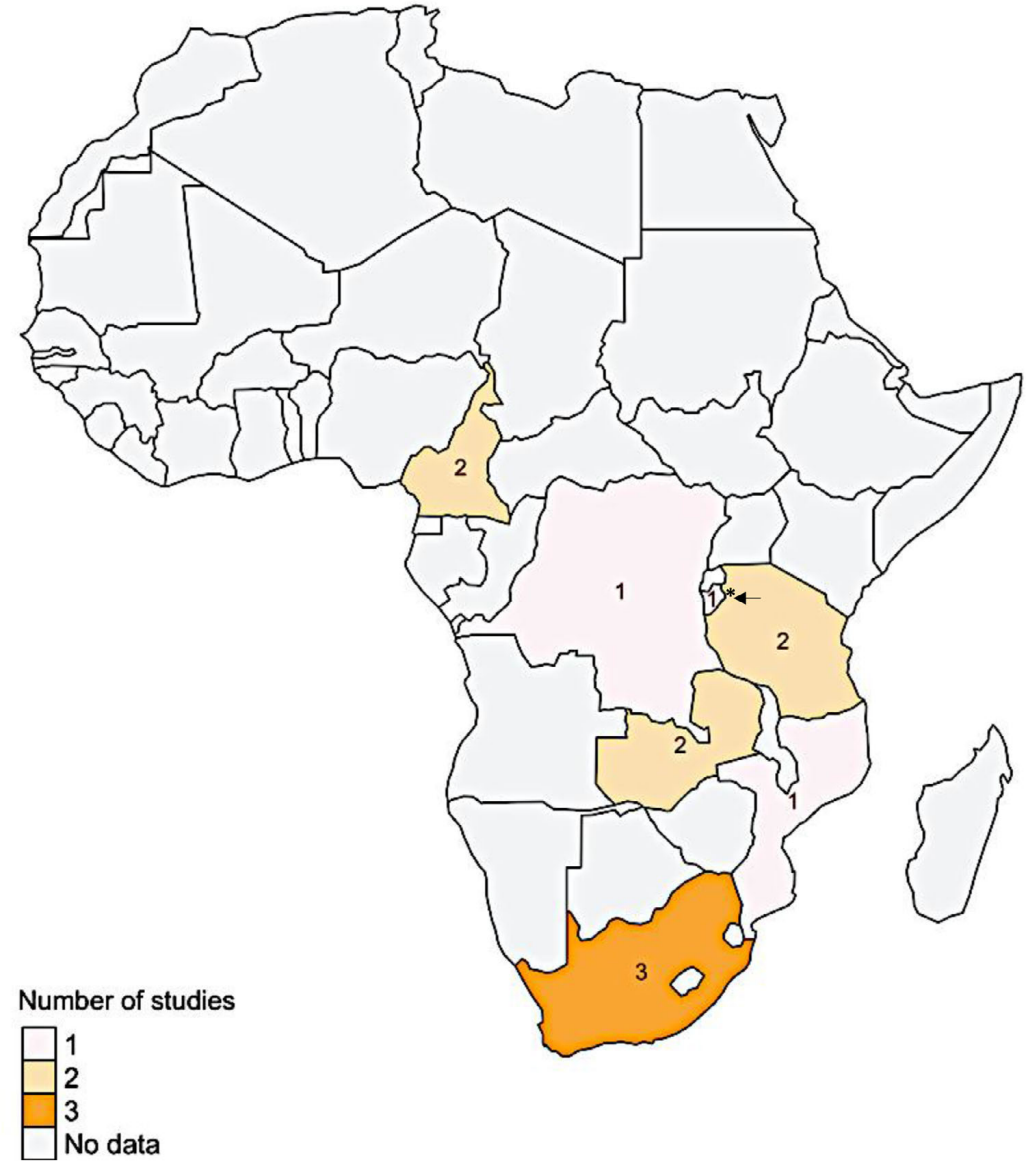
**Map of sub‐Saharan Africa showing geographical locations of studies included in the systematic review**. *Burundi is a small country sharing the southern border with Tanzania (black arrow).

A total of 323 papers were initially identified, with 256 from PubMed and 67 from Scopus. After removing 57 duplicates, we screened the abstracts of the remaining 266 papers (Figure S1). Out of these, 52 papers (Table S4) met the eligibility criteria for full‐text retrieval. The exclusions primarily stemmed from publications that did not pertain to infant ART initiation coverage (*n* = 165), inappropriate study designs such as reviews, randomized cluster trials and clinical trials (*n* = 18), or irrelevant outcomes (*n* = 13). Subsequently, during the full‐text review of the 52 selected papers, 40 were further excluded due to ineligible outcomes or an inability to extract pertinent insights. Consequently, 12 papers remained eligible for data extraction and insight synthesis. Most papers from a single country originated from South Africa (three papers) and retrospective studies (including secondary data analysis) constituted the largest portion of the selected papers (*n* = 6). A detailed breakdown of the included articles and their geographical locations are presented in Table 1 and Figure 1, respectively.

**Table 1:** A detailed breakdown of the studies included in the narrative synthesis

ID	Author	Country	Study design	Study population
1	Jolly et al. [16]	Eswatini	Retrospective case‐control study	HIV‐positive children 2–18 months (*n* = 210 participants, 70 cases and 140 controls)
2	Frigati et al. [17]	South Africa	Retrospective audit study	0–4 weeks years (*n* = 997)
3	Chiduo et al. [18]	Tanzania	Retrospective audit study	HIV‐exposed infants (*n* = 4860)
4	Sutcliffe et al. [19]	Zambia	Longitudinal study	All treatment‐naive children younger than 15 years initiating ART (*n* = 200)
5	Nydal et al. [20]	Tanzania	Retrospective cohort study	Mother‐infant pairs (*n* = 167, but infants were 172 because two mothers had two pregnancies and two had twins)
6	Cook et al. [21]	Mozambique	Secondary data analysis	Mothers living with HIV and their infants (*n* = 443)
7	Tembo et al. [22]	Zambia	Qualitative study	20 In‐depth interviews with a purposive sample of healthcare workers, study staff and caregivers in high‐risk MIPs at six health facilities included in a larger implementation research study evaluating the community POC model
8	Mavedzenge et al. [23]	Burundi, Cameroon and the Democratic Republic of Congo	Secondary data analysis	IeDEA consortium data from surviving children and adolescents, defined as aged 2–17 years (*n* = 404 children, including 25% of the cohorts were 2–4 years)
9	Penda et al. [24]	Cameroon	Descriptive cross‐sectional study	Healthcare providers (*n* = 103)
10	Millar et al. [25]	South Africa	Observational study	Infants acquiring HIV in utero (*n* = 151)
11	Spooner et al. [26]	South Africa	Baseline situational analysis	HIV‐exposed infants for birth PCR testing in hospital (*n* = 323)
12	Ahmed et al. [27]	Eswatini	Qualitative study	Four focus group sessions were held, two with 20 caregivers of ART‐enrolled children (10 in each FGD session) and two with a total of 14 caregivers of non‐enrolled children (one with six and the other with eight caregivers).

Abbreviations: ART, antiretroviral therapy, FGD, focus group‐discussion; IeDEA, International Epidemiology Database to Evaluate AIDS.

### 3.1 Barriers to early infant initiation on ART in SSA

**Figure 2:**
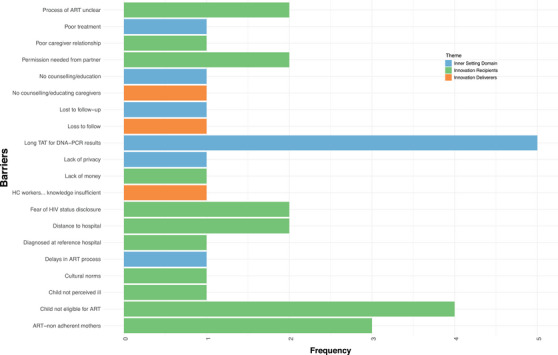
**Histogram showing barriers to ART initiation in infants**. Abbreviations: ART, antiretroviral therapy; TAT, turnaround time; TB, tuberculosis. *turnaround time, antiretroviral therapy, tuberculosis.

The barriers to early infant initiation on ART in SSA can be categorized into two overarching domains within the CFIR (Table 2 and Figure 2): “the inner setting” and “the people involved.” Within the “inner setting” domain, three distinct sub‐themes have emerged: lack of resources for activities and shared beliefs of the society (culture). Within the “people involved” domain, two distinct sub‐themes have come to the fore: mothers of infants exposed to HIV play a pivotal role in the early initiation of ART (innovation recipients). Healthcare providers represent a critical link in the chain of early infant ART initiation (innovation deliverers).

**Table 2:** Barriers to ART initiation in infants

Theme	Sub‐theme	Example	Number of papers reported: Reference
Inner setting domain	Lack of resources	Low human resource leading to long TAT for DNA‐PCR results	5: [18, 19, 20, 26, 27]
		Clients lost to follow‐up due to lack of follow‐up mechanisms	1: [19]
	Culture	Lack of privacy and confidentiality	1: [27]
		Poor treatment in health facilities	1: [27]
		Delays in ART initiation process	1: [27]
	Attitude and conduct	Unavailability of counselling and education	1: [27]
Innovation recipients	Lack of capability	Did not understand the process of ART enrolment	2: [16, 27]
		Permission from their partner before initiating the child on ART	2: [16, 27]
		Child perceived as not being ill	1: [23]
	Motivation restriction	Feared disclosure of the child's HIV status	1: [16]
		Cultural norms, expectations and values	1: [27]
		Poor relationship with caregiver	1: [27]
		Diagnosed at a reference hospital	1: [23]
		Mothers who themselves are ART‐non‐adherent	3: [17, 18, 25]
	Lack of opportunity	Lack of money	1: [27]
		Child was not eligible for ART	4: [16, 18, 27]
		Child just been initiated on TB treatment	[16, 18, 27]
		Child died before initiating on ART	[16, 27]
		Distance to the hospital	^2:^ [16]
Innovation deliverers	Lack of capability	Healthcare workers’ insufficient knowledge on initiating ART	^1:^ [23]
	Lack of opportunity	Loss to follow‐up	1: [18]
	Lack of motivation	Healthcare providers not counselling and educating caregivers	1: [27]

Abbreviations: ART, antiretroviral therapy; TAT, turnaround time; TB, tuberculosis_._

### 3.2 Challenges within the health facility

Table 2 and Figure 2 detail the potential barriers associated with early infant initiation on ART in SSA in the inner setting domain theme. Five publications, two from Tanzania [18, 20], one from Zambia [19], one from Eswatini [27] and one from South Africa [26], reported lack of resources as a barrier. This includes lack of human resources and lack of laboratory resources. In return, these barriers lead to an increase in the TAT for the caregivers to receive DNA‐PCR results of their infants. Of note, only one study by Spooner et al. [26] provided further insights in the context of an implementation study of POC testing. Another study by Chiduo et al. [18] reported a lack of hospital resources to trace the clients which leads to “lost to follow‐up.” The study from Zambia [19] was most different from the rest because it reported barriers to resources in the context of community‐based POC testing.

The second sub‐theme within the health facility was the shared beliefs of the society (culture) from the infant caregiver's perspective, only one qualitative study paper from Eswatini [27] reported on this sub‐theme with several insights. For instance, one caregiver expressed frustration with the health system, citing long wait times and unaccommodating staff schedules:

*“Sometimes you get there at 7 or 6 and the lines are too long. Then at 10 they leave you waiting and go for lunch… Even at 1 they also leave and go to lunch.”*

Another caregiver noted the lack of action and support from health workers, which often led to a feeling of helplessness:

*“…sometimes you wait for more than 2 hours, and the nurses would not help you until it suits them.”*

Furthermore, the violation of privacy and confidentiality at health facilities was a serious concern, as one caregiver's experience of a nurse disclosing their HIV status to others indicates a breach of trust:

*“…there was a nurse who used to tell other visiting patients about my status.”*

The study also unearthed issues of discrimination within the community that deterred individuals from seeking treatment:

*“When we see someone queuing to take ARVs we start asking people if they have seen so and so… the problem is that people start discriminating based on status, so people get afraid to enroll on treatment.”*

Additionally, a lack of counselling for HIV‐positive mothers at clinics, impacting the management of their health and that of their babies, was identified:

*“At the clinic I was attending, I was never counselled on how to take care of myself and my baby when you are HIV positive…”*

The third sub‐theme addressed the attitude and conduct of healthcare workers, which plays a critical role in patient experience and treatment adherence:

*“I think finding people of the right attitude, who are approachable, is very important to us when we come to the health facilities. When you get ill‐treated by a person at the Health Centre, you get demotivated even to come back for refills just because the image of that person who treated you badly comes to mind. You start thinking twice.”*

And another woman from Eswatini stated:

*“We are not treated well; you sometimes wish to change facility and go somewhere where you will be welcomed and free to talk about whatever is troubling you. It's like they are in a hurry when attending patients. They don't have time to sit down and talk to you”* [27].

Noteworthy, the last two themes also contribute to the insights of the innovation recipients and deliverers.

### 3.3 Challenges with the people involved

Table 2 and Figure 2 also show views on the barriers of the people involved in the ART initiation process of the infants living with HIV (healthcare providers and the infant's caregivers).

#### 3.3.1 Barriers in innovation recipients

For the innovation recipients’ sub‐theme, the infants’ caregivers reported “lack of capability” in that they did not understand the process of ART enrolment. The findings from two papers, qualitative [27] and quantitative [16] evaluation by the same team from Eswatini, held different views on whether the long distance is a barrier or enabler for ART initiation coverage. The qualitative paper reported that the mothers reported distance coupled with lack of transport as a barrier to ART initiation. The quantitative paper found that infants who lived ≤ 30 minutes from the health facility were 84% less likely to initiate on ART as compared to those who lived >60 minutes away (statistically significant). Further, the authors discussed that mothers preferred to travel to distant health facilities in fear of stigma. While addressing the same sub‐theme, it was observed that women often did not possess the autonomy to begin ART for their child without the consent of their spouse or partner:

*“…..6% reported that they had to get permission from their partner before initiating the child on ART”* [16].

The infants’ mothers also reported “motivation‐restrictions” such as fear of disclosing the child's status due to stigma and distance to the hospital as barrier (*n* = 2, both from Eswatini). Three papers (*n* = 2 from South Africa and *n* = 1 from Tanzania) reported that mother's non‐adherent to ART were demotivated to commence their infant on ART. One paper from Eswatini reported the following as motivation barriers in initiating infants on ART; poor rapport between healthcare providers and the caregiver, caregiver's denial, caregiver's guilt; and an infant with no opportunistic infections (and hence infant not requiring immediate healthcare).

One paper, which reports data from Burundi, Cameroon and the Democratic Republic of Congo [23], reported several factors that may impede the mother initiating their infant on ART. The authors report that mothers aware of their that they are living with HIV were less likely to have an infant initiated on ART. It also reported that being told that their infant was living with HIV at a tertiary hospital compared to a primary health facility was associated with demotivation of commencing infant on ART:

*“Diagnosis at a reference hospital was significantly associated with not having initiated ART……..”*

Some mothers reported unfavourable circumstances as a barrier. For example, when the infant has also been diagnosed with tuberculosis (TB) and commenced on TB therapy, they should not commence on ART. This guideline is to potentially mitigate adverse side effects for the medication interactions for an immature immune system (*n* = 2 from Eswatini and *n* = 1 from Tanzania). One woman from Eswatini also reported a lack of money as a lack of opportunity to commence infant on ART,

*“I think it is because of lack of money, because I can fetch the medication but if we do not have enough food to go with the medication, it becomes a problem. Because once I initiate him on treatment, he must not stop taking it, so it's better to not initiate the child on treatment.”*

#### 3.3.2 Barriers in innovation deliverers

One paper from Cameroon [24] reported that healthcare providers lacked the necessary skills and knowledge to initiate infants. The paper further states that their knowledge on first‐line ART protocols was insufficient, due to little information about standard procedures. In other instances, the healthcare providers reported unfavourable circumstances (lack of opportunity) to commence infants on ART because the infant did not report back to the assigned date at the clinic to attain their results and hence reported as a “lost to follow‐up” (*n* = 1, Tanzania).

### 3.4 AACTT mapping of the concepts of barriers to early infant initiation to implementation strategies

Table 3 is a behavioural specification framework called the AACTT framework. It was used to map the barriers identified to implementation strategies. It shows that acquiring adequate resources (human, cartilages and POC testing) a suggested strategy for the barrier of long TAT to receive POC testing results (*n* = 5); therefore, acquiring adequate resources (human, cartilages and POC testing). In addition, implementing community POC testing would be a probable implementation strategy. The people responsible for these activities would be the hospital governance, management team and Implementation science scientist. Another concept of strategy mapping is that the unmet needs of the mother and father of the infants living with HIV can be addressed by healthcare professionals, partners and peers (peer support) in hospitals and community throughout the VT cascade and beyond. The challenge of insufficient knowledge and skills of healthcare workers and practices regarding initiating ART in children less than 2 years and first‐line ART protocols including TB treatment protocol can be addressed by trainings including developing work plans around infants on TB treatment at 6 weeks–24 months in hospitals.

**Table 3:** AACTT mapping of the concepts of barriers to early infant initiation to implementation strategies

Barrier	Action: A discrete observable behaviour	Actor: The individual or group of individuals who perform (or should/could) the Action	Context: The physical, emotional or social setting in which the Actor performs (or should/could) the Action	Target: The individual or group of individuals for/with/on behalf of whom the Actor performs the Action	Time: The time period and duration that the Actor performs the Action in the Context with/for the Target
Lack of resources for activities	Acquire adequate resources (human, cartridges and point‐of‐care testing)	The hospital governance and management team	Care points and Laboratory	Laboratory team	Ongoing
Long distance to the hospital for the caregivers	Pilot community point‐of‐care testing	The hospital governance, management team and Implementation Science scientist	Research Institutions	The caregiver of the infant exposed to HIV	Study period
Mothers who themselves are ART‐non‐adherent Permission from their partner before initiating the child on ART Cultural norms, expectations and values	Address unmet needs of the mother and father	Healthcare professionals, partners and peer support	Hospitals and community and Research Institutions	The caregiver of the infant exposed to HIV	Throughout the VT cascade and beyond
Healthcare workers’ insufficient knowledge and skills on initiating ART in infants less than 2 years and first‐line ART protocols Unavailability of counselling and education	Develop training and behavioural change strategies	The hospital governance and management team	Hospitals	The healthcare professionals and support staff	Ongoing
Child was not eligible for ART due to TB diagnosis	Develop work plan around TB treatment	The healthcare professionals and support staff	Hospitals	The healthcare professionals and support staff	6 weeks–24 months

Abbreviations: AACT, Action‐Actor‐Context‐Target‐Time; ART, antiretroviral therapy; TB, tuberculosis; VT, Vertical Transmission.

### 3.5 Quality assessment

We assessed the quality of evidence of the qualitative papers using the 10 aspects of the CASP checklist listed in Table S5. Of the two qualitative papers included in this systematic review, the only limited aspect of rigour was an explanation of the relationship between the researcher and participants. Both papers did not include a section on reflexivity and were rated down for this. Figure S2 summarizes the completeness of reporting using the STROBE checklist, including the title, introduction, methodology, results, discussion and funding. Colour coding indicates whether reporting was complete (green), partially reported (yellow) or missing (red). Of the criteria assessed, generalizability was the least well‐reported methods criteria (8/10 [80%]). Other criteria that should have been reported more comprehensively were the interpretation, abstract, bias and other analyses. However, the main study results of interest for this review were clear and detailed for all included studies.

## 4 DISCUSSION

This narrative synthesis focuses on the identification of barriers and potential strategies for early infant initiation of ART in SSA. While efforts are being made to improve early infant initiation with POC testing, challenges persist. We identified structural barriers, namely health system, social and cultural. We highlight the impact of limited human and laboratory resources on the time it takes to receive DNA‐PCR results and extend ART initiation coverage. Additionally, we emphasize the significance of male involvement and comprehensive healthcare provider training (behavioural, skills and social). We also delve into key areas for investment, such as human and laboratory resources POC testing, implementation strategies, and peer support for infant mothers and their partners. Furthermore, we stress the importance of training healthcare professionals in managing infants living with HIV. Some of our findings align with recent literature related to the topic, validating our approach [28, 29, 30, 31].

For example, POC testing has been shown to improve ART initiation by reducing the TAT of test results [11, 13, 32]. However, a challenge comes in the context of the high burden of disease like in South Africa, where some clients are not provided with the same‐day testing and initiation due to a large ART cohort and a small number of service providers. This situation may lead to a loss of follow‐up of clients. However, this is when a DBS‐sample collection protocol is used. Notably, the TAT of the receipt of DNA‐PCR results has been shown to be reduced to as low as 5.34 hours when Xpert HIV is used with a WB protocol [11, 33]. In addition, it is shown to be cost‐minimizing and acceptable to most caregivers, but the biggest concern was the amount of blood (thought to be excess) withdrawn from the infant [33]. Community POC testing on high‐risk mother‐infant pairs has also been explored in Zambia, and the results show that it minimizes the structural barriers to EID testing access. However, operational challenges such as client's confidentiality needs to be addressed [34]. We, in Malawi, recently showed that in a busy HIV care clinic, simple strategies (such as tagging of care mastercards), developed by its team using the model of improvement, improved the number of infants receiving their DNA‐PCR results (unpublished). Future studies may assess which strategy is more cost‐effective among “resources to boost the current work plan through quality improvement projects,” “the whole blood Protocol for POC testing” and “Community POC testing.”

In terms of the unmet needs of the mother, our narrative systematic review demonstrates that mothers of infants who get support from their partners are more likely to be retained on the VT cascade and bring their child for testing and initiation [35, 36, 37]. Another systematic review of the VT cascade [38] also showed that male partner involvement in VT reported reductions in infant HIV acquisition of up to (RR 0.61; 95% CI 0.39–0.94, I^2^ = 0%). However, in Malawi, improvement of male participation in antenatal care using word of mouth or an invitation card was assessed as a strategy for improving male involvement [39]. Notably, most of the women (66.5%) returned to the clinic without a male partner, and there were no meaningful differences between the two groups. However, male involvement is a key factor in the uptake of EID of HIV and retention in care [40], therefore, further investments in a refined theory and mechanism are essential to address the unmet needs of the infant's mother. This same review [38] also reported that mobile phone‐based interventions showed a statistically significant increase (pooled RR 1.18; 95% CI 1.05–1.32, I^2^ = 83%) in uptake of EID of HIV at around 6 weeks postpartum. However, we have assessed short text messaging reminders as a strategy. We found that in rural Malawi this strategy showed no improvement in the retention of infants, and it was also associated with stigma [41].

To the best of our knowledge, these are the best available insights on the topic of barriers and potential strategies for early infant initiation on ART. Some concepts such as TAT between testing and receipt, TB treatment, non‐adherent mothers and lack of psychological capability (knowledge) agree in two or more studies. This systematic review also identifies gaps in strategies that work but need improving. Finally, it identifies areas that can improve early infant initiation but have not been investigated yet, such as the effect of training healthcare providers on guidelines for HIV‐exposed infants care management. This systematic review searched PUBMED and Scopus. By searching only these two databases, we might have missed some papers; however, these two databases are known for comprehensive coverage across various disciplines, including HIV research. This study excluded studies reporting purely on early infant diagnosis and other components of the VT cascade, independent systems that are interconnected to early infant initiation. Therefore, some insights may have been missed. However, by presenting factors affecting early infant initiation on ART, this systematic review offers valuable insights that can inform strategies in analogous settings (transferability). For example, if a particular insight was from Eswatini only, countries with similar early infant initiation processes may reinforce that insight's relevance and practical applicability. This systematic review is one of a series of projects documenting the development and evaluation of interventions to improve ART initiation in infants before 12 weeks [33, 42]. Notably, there is a paucity of insights on this topic.

## 5 CONCLUSIONS

This narrative systematic review suggests that investments in timely resources for POC testing operations, the unmet needs of the infants’ mothers and training of care workers may improve timely ART initiation in infants. However, there are currently mixed findings on the effectiveness of these strategies in different SSA contexts. Therefore, future studies should further explore these interventions’ theories of action and mechanism.

## COMPETING INTERESTS

The authors declare no competing interests.

## AUTHORS’ CONTRIBUTIONS

VM is the guarantor of this manuscript. VM, MCH, CIJC and MNN developed the research question. CIJC, VM and NM devised the search strategy. CIJCC and DS performed data extraction and quality assessments of the papers. CIJC, DS and ALN‐M performed the data analysis. MM, MKK and AC made substantial contributions to the study design. CIJC drafted the manuscript. All authors have read and approved the final manuscript.

## FUNDING

Research reported in this publication was supported by the Fogarty International Center of the National Institutes of Health under Award Number D43TW010060. The content is solely the responsibility of the authors and does not necessarily represent the official views of the National Institutes of Health.

## CME STATEMENT

This article is published as part of a supplement supported by unrestricted educational grant by ViiV Healthcare.

Credits Available for this Activity: American Medical Association (AMA Credit).

Washington University School of Medicine in St. Louis designates this enduring material for a maximum of 1 AMA PRA Category 1 Credit™. Physicians should claim only the credit commensurate with the extent of their participation in the activity.

## Supporting information


**Table S1**: Search Strategy of the systematic narrative synthesis
**Table S2**: PICOS Systematic Review Framework
**Table S3**: Inclusion and Exclusion Criteria
**Table S4**: 52 papers for full text review for the systematic narrative synthesis
**Table S5**: Critical Appraisal Skills Programme (CASP) checklist report for the qualitative papers included in the systematic narrative synthesis

**Figure S1**: PRISMA diagram for study selection flow
**Figure S2**: Quality assessment of the observational studies included in the narrative synthesis using the STROBE checklist

## Data Availability

Data are available upon request.
