# Supplementary material for: Examining barriers to antiretroviral therapy initiation in infants living with HIV in sub‐Saharan Africa despite the availability of point‐of‐care diagnostic testing: a narrative systematic review
Source: J Int AIDS Soc. 2024 Jul 5;27(Suppl 1):e26284. doi: 10.1002/jia2.26284 (PMC11224580; doi:10.1002/jia2.26284)
Supplement: Supplementary file 1 — Table S1: Search Strategy of the systematic narrative synthesis Table S2: PICOS Systematic Review Framework Table S3: Inclusion and Exclusion Criteria Table S4: 52 papers for full text review for the systematic narrative synthesis Table S5: Critical Appraisal Skills Programme (CASP) checklist report for the qualitative papers included in the systematic narrative synthesis [file JIA2-27-e26284-s002.docx]

TS1: Search Strategy of the systematic narrative synthesis

| **Search Number** | **Keyword** | **Query** | **Results** | **Date and time of the search** |
| --- | --- | --- | --- | --- |
| 1. | "Antiretroviral therapy" OR "ART" OR  "ART Initiation" | "Antiretroviral therapy"[All Fields] OR "ART"[All Fields] OR "ART Initiation"[All Fields] | 203,111 | 18^th^ January 2023, 22:04:17 |
| 2. | "Sub-saharan Africa" | "sub sahara*"[All Fields] AND ("africa"[MeSH Terms] OR "africa"[All Fields] OR "africa s"[All Fields] OR "africas"[All Fields])) OR ("africa, western"[MeSH Terms] OR ("africa"[All Fields] AND "western"[All Fields]) OR "western africa"[All Fields] OR ("west"[All Fields] AND "africa"[All Fields]) OR "west africa"[All Fields]) OR ("africa, eastern"[MeSH Terms] OR ("africa"[All Fields] AND "eastern"[All Fields]) OR "eastern africa"[All Fields] OR ("east"[All Fields] AND "africa"[All Fields]) OR "east africa"[All Fields]) OR ("africa, central"[MeSH Terms] OR ("africa"[All Fields] AND "central"[All Fields]) OR "central africa"[All Fields] OR ("central"[All Fields] AND "africa"[All Fields])) OR ("south*"[All Fields] AND ("africa"[MeSH Terms] OR "africa"[All Fields] OR "africa s"[All Fields] OR "africas"[All Fields])) OR "SSA"[All Fields] OR ("cameroon"[MeSH Terms] OR "cameroon"[All Fields] OR "cameroons"[All Fields] OR "cameroon s"[All Fields]) OR "Central African Republic"[All Fields] OR ("chad"[MeSH Terms] OR "chad"[All Fields]) OR ("congo"[MeSH Terms] OR "congo"[All Fields]) OR "Democratic Republic of the Congo"[All Fields] OR ("gabon"[MeSH Terms] OR "gabon"[All Fields]) OR (("burundi"[MeSH Terms] OR "burundi"[All Fields]) AND ("djibouti"[MeSH Terms] OR "djibouti"[All Fields])) OR ("eritrea"[MeSH Terms] OR "eritrea"[All Fields]) OR ("ethiopia"[MeSH Terms] OR "ethiopia"[All Fields] OR "ethiopia s"[All Fields]) OR ("kenya"[MeSH  Terms] OR "kenya"[All Fields] OR | 702,685 | 18^th^ January 2023, 22:00:38 |

|  |  | "kenya s"[All Fields]) OR ("rwanda"[MeSH Terms] OR "rwanda"[All Fields] OR "rwanda s"[All Fields]) OR ("somalia"[MeSH Terms] OR "somalia"[All Fields]) OR ("sudan"[MeSH Terms] OR "sudan"[All Fields] OR "sudans"[All Fields] OR "sudan s"[All Fields]) OR ("tanzania"[MeSH Terms] OR "tanzania"[All Fields] OR "tanzania s"[All Fields]) OR ("uganda"[MeSH Terms] OR "uganda"[All Fields] OR "uganda s"[All Fields]) OR ("angola"[MeSH Terms] OR "angola"[All Fields] OR "angola s"[All Fields]) OR ("botswana"[MeSH Terms] OR "botswana"[All Fields] OR "botswana s"[All Fields]) OR ("lesotho"[MeSH Terms] OR "lesotho"[All Fields]) OR ("malawi"[MeSH Terms] OR "malawi"[All Fields] OR "malawi s"[All Fields]) OR ("mozambique"[MeSH Terms] OR "mozambique"[All Fields] OR "mozambique s"[All Fields]) OR ("namibia"[MeSH Terms] OR "namibia"[All Fields] OR "namibia s"[All Fields]) OR ("south africa"[MeSH Terms] OR ("south"[All Fields] AND "africa"[All Fields]) OR "south africa"[All Fields]) OR ("eswatini"[MeSH Terms] OR "eswatini"[All Fields] OR "swaziland"[All Fields]) OR ("zambia"[MeSH Terms] OR "zambia"[All Fields] OR "zambia s"[All Fields]) OR ("zimbabwe"[MeSH Terms] OR "zimbabwe"[All Fields] OR "zimbabwe s"[All Fields]) OR ("benin"[MeSH Terms] OR "benin"[All Fields] OR "benin s"[All Fields]) OR ("burkina faso"[MeSH Terms] OR ("burkina"[All Fields] AND "faso"[All Fields]) OR "burkina faso"[All Fields]) OR ("cabo verde"[MeSH Terms] OR ("cabo"[All Fields] AND "verde"[All Fields]) OR "cabo verde"[All Fields] OR ("cape"[All Fields] AND "verde"[All Fields]) OR "cape verde"[All Fields]) OR ("cote d ivoire"[MeSH Terms] OR ("cote"[All Fields] AND "d ivoire"[All Fields]) OR "cote d ivoire"[All Fields]) OR ("gambia"[MeSH Terms] OR "gambia"[All Fields] OR "gambia s"[All Fields]) OR ("ghana"[MeSH Terms] OR "ghana"[All Fields] OR "ghana s"[All Fields]) OR  ("guinea"[MeSH Terms] OR |  |  |
| --- | --- | --- | --- | --- |

|  |  | "guinea"[All Fields] OR "guinea s"[All Fields] OR "guineas"[All Fields]) OR ("equatorial guinea"[MeSH Terms] OR ("equatorial"[All Fields] AND "guinea"[All Fields]) OR "equatorial guinea"[All Fields]) OR ("guinea bissau"[MeSH Terms] OR "guinea bissau"[All Fields] OR ("guinea"[All Fields] AND "bissau"[All Fields]) OR "guinea bissau"[All Fields]) OR ("liberia"[MeSH Terms] OR "liberia"[All Fields] OR "liberia s"[All Fields]) OR ("mali"[MeSH Terms] OR "mali"[All Fields]) OR ("mauritania"[MeSH Terms] OR "mauritania"[All Fields]) OR ("niger"[MeSH Terms] OR "niger"[All Fields]) OR ("nigeria"[MeSH Terms] OR "nigeria"[All Fields] OR "nigeria s"[All Fields]) OR ("senegal"[MeSH Terms] OR "senegal"[All Fields] OR "senegal s"[All Fields]) OR ("sierra leone"[MeSH Terms] OR ("sierra"[All Fields] AND "leone"[All Fields]) OR "sierra leone"[All Fields]) OR ("togo"[MeSH Terms] OR "togo"[All Fields]) OR ("comoros"[MeSH Terms] OR "comoros"[All Fields] OR "comoro"[All Fields]) OR ("madagascar"[MeSH Terms] OR "madagascar"[All Fields] OR "madagascar s"[All Fields]) OR ("mauritius"[MeSH Terms] OR "mauritius"[All Fields]) OR ("seychelles"[MeSH Terms] OR "seychelles"[All Fields]) OR ("melanesia"[MeSH Terms] OR "melanesia"[All Fields] OR ("solomon"[All Fields] AND "islands"[All Fields]) OR "solomon islands"[All Fields]) OR ("micronesia"[MeSH Terms] OR "micronesia"[All Fields] OR ("marshall"[All Fields] AND "islands"[All Fields]) OR "marshall islands"[All Fields]) OR "Sao Tome  AND Principe"[All Fields] |  |  |
| --- | --- | --- | --- | --- |
| 3. | "Barriers" and "Enablers" | "Challenges"[All Fields] OR "Barriers"[All Fields] OR "Facilitators"[All Fields] OR "Enablers"[All Fields] | 577,329 | 18^th^ January 2023, 22:07:49 |
| 4 | "Infant" | "infant"[MeSH Terms] OR "infant"[All Fields] OR "infants"[All Fields] OR "infant s"[All Fields] | 1,360,916 | 18^th^ January 2023, 21:52:33 |

**TS2: PICOS Systematic Review Framework**

| **Population** | “Infants exposed to HIV” or people living with HIV (0-12 months demographic) from SSA |
| --- | --- |
| **Exposure** | Initiation on ART |
| **Control** | N/A |
| **Outcome** | Barriers and Challenges |
| **Study Design** | Observational study, Qualitative study, and Mixed Method Studies |

**Table S3: Inclusion and Exclusion Criteria**

| **Inclusion criteria** | **Exclusion criteria** |
| --- | --- |
| 1. Qualitative, Cross-sectional, Cohort, longitudinal and mixed research designs | 1. Studies reporting on the other components of the VT cascade; Antenatal care visit, HIV test of the mother, Receipt of results of the mothers and Initiation of ART prophylaxis of the mother |
| 1. Studies that reported influences (positive or negative) of early infant initiation on ART | 1. Studies reporting insights on influencers of early infant initiation on ART not from SSA |
| 1. Studies published in peer-reviewed journals | 1. Randomized “Control” Clinical Trial designs |
| 1. Studies from SSA | 1. Studies reporting on Early Infant Diagnosis |
|  |  |

**TS4: 52 papers for full text review for the systematic narrative synthesis**

| **Study ID** | **Author** | **Study Site** | **Study Design** | **Study Population** |
| --- | --- | --- | --- | --- |
| 1 | Katirayi et al. 2022 | Zimbabwe | Qualitative | Caregivers |
| 2 | Rencken et al. 2022 | South Africa | Cross sectional | Infants |
| 3 | Karugaba et al. 2022 | Bostwana | Qualitative | Fifteen FGDs with 142 mothers (aged 21–52 years) |
| 4 | Jolly et al. 2018 | Swaziland | Retrospective case-control study | Children 2-18 months living with HIV |
| 5 | Frigati et al. 2017 | South Africa | Retrospective audit study | 0-4 weeks infants exposed to HIV, N=997 |
| 6 | Chiduo et al. 2015 | Tanzania | Retrospective audit study | 4,860 infants exposed to HIV |
| 7 | Sutcliffe et al. 2016 | Zambia | Longitudinal study | N=200 of all treatment-naive children younger than 15 years initiating ART |
| 8 | Castelnuovo et al 2017 | Uganda | Before and after | N= 700 pregnant women |
| 9 | Sinunu et al. 2014 | Malawi | Survey | N=5,634 caregiver-infant pairs |
| 10 | Ugwu et al. 2013 | Nigeria | A cross-sectional survey | N=280 children child-mother pairs |
| 11 | Carlberg et al.2022 | Ethiopia | Prospectively data collection | N=231 children |
| 12 | Napyo et al. 2020 | Uganda | Prospective cohort study | N=472 mother-infant pairs |
| 13 | Nydal et al. 2021 | Tanzania | Retrospective cohort study | N=167 mother infant pairs (172 because two mothers had 2 pregnancies and two had twins |
| 14 | Kikuchi et al. 2014 | Rwanda | Qualitative | N=121 caregivers of children living with HIV in Kigali |
| 15 | Cook et al. 2012 | Mozabique | Logistic regression | N=443 HIV-infected mothers and their infants |
| 16 | Tembo et al. 2022 | Zambia | Qualitative | N=20 Caregivers |
| 17 | Dijk et al. 2009 | Zambia | cross-sectional analysis | N=192 children living with HIV |
| 18 | Nuoh et al. 2020 | Ghana | An unmatched case control study | N=96 cases and 96 controls |
| 19 | Bergmann et al. 2017 | Uganda | Mixed methods study | N=Cross-sectional survey (n = 384) and focus group discussions (n = 6, 5–9 participants each) |
| 20 | Mavedzenge et al 2016 | Burundi, Cameroon, and the Democratic Republic of Congo | Secondary data analysis | N=2–17 years of age attending HIV programs in Central Africa. Programmatic data from 404 children |
| 21 | Penda et al 2019 | Cameroon | Descriptive cross-sectional study | N=103 health care providers |
| 22 | Baryamutuma et al. 2017 | Uganda | Cross-sectional assessment | N=505 health care facilities |
| 23 | Milar et al. 2020 | South Africa | observational study | Infants acquiring HIV in utero (n=151) |
| 24 | Buesseler et al. 2014 | Côte d'Ivoire | Semistructured interviews | N=24 women living with HIV |
| 25 | Ssanyu et al. 2020 | Uganda | Cross-sectional study | caregivers of 206 children under 5 years living with HIV who were attending health facilities in Jinja and had been on ART for at least 3 months. |
| 26 | Spooner et al. 2019 | South Africa | Baseline situational-analysis | Infants exposed for birth PCR testing in hospital (N = 323) |
| 27 | Teasdale et al. | South Africa | Prerandomization phase of a clinical trial | N=269 children |
| 28 | Wexler et al 2019 | Kenya | Qualitative | N=26 HIV care providers and 35 parents of infants exposed to HIV (including 23 mothers, 6 fathers, and 3 mother-father pairs) at four study |
| 29 | Kabue et al. 2012 | Malawi, Lesotho, and Swaziland. | Retrospective cohort study | N=2306 patients |
| 30 | Adedimeji et al. 2017 | Central Africa | Secondary data analysis | N=3,426 children (0–15 years) |
| 31 | Gill et al. 2018 | Lesotho | Observational prospective cohort | N=20 women, 18 HWs and 9 district/central laboratory staff |
| 32 | Jones et al. 2019 | Malawi, South Africa and United Republic of Tanzania | Qualitative | N=400,000 |
| 33 | Coulibaly et al. 2014 | Burkina Faso | Qualitative | N=67,592 |
| 34 | Myer et al. 2016 | South Africa | 3 interrelated phases with observational and experimental elements, | N=1600 |
| 35 | Mutanga et al. 2020 | Zambia | Secondary data analysis | N=2630 children |
| 36 | Ahmed et al. 2018 | Swaziland | Qualitative data | Four focus group sessions were held, two with 20 caregivers of ART-enrolled children (10 in each FGD session) and two with a total of 14 caregivers of non-enrolled children (one with 6 and the other with 8 caregivers). |
| 37 | Adeyinka et al 2017 | Western Africa (Cote d’Ivoire, Ghana and Nigeria), Central Africa (Angola, Cameroon, Chad, and Congo), Eastern Africa (Burundi, Ethiopia, Kenya, and Uganda), and Southern Africa (Botswana, Lesotho, Malawi, Mozambique, Namibia, South Africa, Swaziland, Tanzania, Zambia, and Zimbabwe). | Ecological analyses | United Nations agencies and Forum for a new World Governance reports for the 21 Global Plan priority countries in Africa with highest burden of mother-to-child HIV transmission. |
| 38 | Kiyaga et al. 2021 | Uganda | Retrospective cohort study | N = 707 |
| 39 | Mohamed et al 2021 | Papua New Guinea | Qualitative substudy | N =18 |
| 40 | Hampanda et al 2017 | Zambia | Cross-sectional survey | 320 HIV-positive mothers who had brought their child for routine |
| 41 | Nyandiko et al | Kenya | Retrospective cohort study | HIV-exposed infants enrolled between February 2002 and July 2007, at any of the United States Agency for International Development-Academic Model Providing Access To Healthcare partnership clinics. |
| 42 | Sengayi et al 2013 | South Africa | Secondary data analysis | 4,266 children |
| 43 | Somi et al 2017 | Tanzania | Secondary data analysis | 29,531 (14,304 boys and 15,227 girls) ART-naive children aged 0-14 years |
| 44 | Kimani-Murage et al 2012 | South Africa | Qualitative | 880 children aged 1–5 years |
| 45 | DiCarlo et al 2018 | Kenya | Qualitative study | 15 lay health workers (Mama Mshauri) |
| 46 | Amani-Bosse et al 2017 | Cote d'Ivoire and Burkina | Prospective therapeutic cohort | 156 children-initiated ART at a median age of 13.9 |
| 47 | Adeniyi et al 2015 | South Africa | Qualitative exploration | Individual interviews (n = 24) Focus group discussion (n = 16) |
| 48 | Gunda et al 2017 | Malawi | Mixed methods study | 20 health facilities implemented MIP clinics. Health care workers' performance implementing MIP clinics was assessed through a mentorship score from 0 to 5 and supplemented with qualitative data from mentorship reports. |
| 49 | Gaitho et al | Kenya | Cross-sectional study | All HEI who had their first HIV virologic test done between January 2015 and December 2017 were included in the study and categorized as either having the test within or after 8 weeks of birth. |
| 50 | Feucht et al 2015 | South Africa | Secondary data analysis | 250 newly diagnosed HIV-infected children |
| 51 | Ciama et al 2012 | Mozambique | Before and after | n=791 |
| 52 | Yeap et al 2010 | South Africa | Qualitative | Children (all demographics) living with HIV, their caregivers (n=21) and health care workers (n=21) |

**TS5: Critical Appraisal Skills Programme (CASP) checklist report for the qualitative papers included in the systematic narrative synthesis**

| Items | Tembo et al. 2022 | Ahmed et al. 2018 |
| --- | --- | --- |
| 1. Was there a clear statement of the aims of the research? | Y | Y |
| 1. Is a qualitative methodology appropriate? | Y | Y |
| 1. Was the research design appropriate to address the aims of the research? | Y | Y |
| 1. Was the recruitment strategy appropriate to the aims of the research? | Y | Y |
| 1. Was the data collected in a way that addressed the research issue? | Y | Y |
| 1. Has the relationship between researcher and participants been adequately considered? | N | N |
| 1. Have ethical issues been taken into consideration? | Y | Y |
| 1. Was the data analysis sufficiently rigorous? | Y | Y |
| 1. Is there a clear statement of findings? | Y | Y |
| 1. How valuable is the research? | Y | Y |
