# Supplementary material for: Examining barriers to antiretroviral therapy initiation in infants living with HIV in sub‐Saharan Africa despite the availability of point‐of‐care diagnostic testing: a narrative systematic review
Source: J Int AIDS Soc. 2024 Jul 5;27(Suppl 1):e26284. doi: 10.1002/jia2.26284 (PMC11224580; doi:10.1002/jia2.26284)
Supplement: Supplementary file 2 — Figure S1: PRISMA diagram for study selection flow Figure S2: Quality assessment of the observational studies included in the narrative synthesis using the STROBE checklist [file JIA2-27-e26284-s001.docx]

FS1: PRISMA diagram for study selection flow^[[1]](#footnote-1)^(1)

**Identification of studies via databases and registers**

Records removed *before screening*:

Duplicate records removed (n = 56)

Records identified from:

Databases (n = 323)

- PUBMED (n = 256)
- Scopus (n = 67)

**Identification**

Records screened

(n = 266)

Records excluded

(n = 214)

Reports sought for retrieval

(n = 52)

Reports not retrieved

(n = 40)

**Screening**

Reports assessed for eligibility

(n = 12)

Studies included in review

(n = 12)

**Included**

^1^PRISMA [Internet]. [cited 2024 Mar 7]. Available from: http://prisma-statement.org/prismastatement/flowdiagram.aspx

FS2: Quality assessment of the observational studies included in the narrative synthesis using the Strengthening the reporting of observational studies in epidemiology (STROBE) checklist^[[2]](#footnote-2)^

1. PR

   [↑](#footnote-ref-1)
2. Key:

   1: Not reported

   2: Some reported

   3: Clear and detailed [↑](#footnote-ref-2)
